# Early changes in cytokine expression in peste des petits ruminants disease

**DOI:** 10.1186/1297-9716-45-22

**Published:** 2014-02-22

**Authors:** Jana Baron, Abdelghani Bin-Tarif, Rebecca Herbert, Lorraine Frost, Geraldine Taylor, Michael D Baron

**Affiliations:** 1 The Pirbright Institute, Ash Road, Surrey GU24 0NF, UK

## Abstract

Peste des petits ruminants is a viral disease of sheep and goats that has spread through most of Africa as well as the Middle East and the Indian subcontinent. Although, the spread of the disease and its economic impact has made it a focus of international concern, relatively little is known about the nature of the disease itself. We have studied the early stages of pathogenesis in goats infected with six different isolates of Peste des petits ruminants virus representing all four known lineages of the virus. No lineage-specific difference in the pathogenicity of the virus isolates was observed, although there was evidence that even small numbers of cell culture passages could affect the degree of pathogenicity of an isolate. A consistent reduction in CD4^+^ T cells was observed at 4 days post infection (dpi). Measurement of the expression of various cytokines showed elements of a classic inflammatory response but also a relatively early induction of interleukin 10, which may be contributing to the observed disease.

## Introduction

Peste de petits ruminants virus (PPRV) is a paramyxovirus that belongs to the genus *Morbillivirus*. It is widely distributed in Africa, the Middle East and Asia, and has a major economic impact on livestock keepers in developing countries, causing a severe disease in small ruminants (sheep and goats), livestock animals that are often the prerogative of the poorer groups in these countries, especially women [1]. The virus has been spreading continuously in recent decades [2], and is beginning to receive significant attention in an effort to control and possibly even eradicate the disease [3,4].

Despite the economic and social importance of PPR as a disease, and a number of recent publications discussing potential new vaccines, the disease itself has not received very close attention. There were extensive descriptions of the clinical signs typical of PPRV infection in the period when the disease was first recognised (reviewed in [5]), as well as descriptions of the identification of the disease in various regions or in specific wildlife species. There have also been reports that, in some cases, specific breeds of goats may be more susceptible than others [6] and that goats may be more susceptible, or develop more severe pathology, than sheep [7-9]. There has been little close study of the progress or causes of pathology, apart from a recent thorough histological investigation of the distribution of the virus during the early stages of infection [10], which showed that the virus spread in a similar way to measles virus spread in humans [11,12], and a study of interleukin 4 (IL-4) and gamma interferon (IFNγ) induction during PPRV infection [13]. In most cases, the studies have used one strain of virus in one host species, making comparisons more difficult. We have carried out a study in goats of the effects of six wild-type isolates, both recent and archived, including representative isolates of all four lineages, examining the clinical picture, the replication of the virus and the effects of the infection on a broad spectrum of cytokines. We found that the clinical picture was not linked to specific lineages, although some isolates were slightly more pathogenic than others. In addition we observed changes in cytokine mRNA levels that matched the expected picture of an inflammatory disease, with the addition of an early increase in IL-10 transcripts, suggesting this cytokine may play a role in the subsequent pathogenic effects observed.

## Materials and methods

### Cells and viruses

The PPRV isolates used (Table 1) were grown and titred on Vero cells expressing the canine signalling lymphocyte activation molecule (SLAM) (Vero-dog-SLAM cells) [14]. Cells, media and foetal calf serum were free from adventitious agents such as pestiviruses. All viruses were archived virus stocks held at the Pirbright Institute Reference Laboratory with the exception of PPRV Kurdistan/2011, which was the kind gift of Dr Bernd Hoffman, FLI, Germany. All the virus stocks used were checked by real-time PCR and shown to be without detectable contamination with any serotype of bluetongue virus (assay carried out by the EU Reference Laboratory for BTV) or any pestivirus (using the pan-pestivirus primers originally described by Vilcek et al. [15]).

**Table 1:** Virus isolates used in the study

**Goats**	**Virus**	**Lineage**	**Passage history**^**1**^
1-3	Kurdistan/2011	4	CVS1 VDS2
4-6	Sudan/Sinnar/72	3	BK2 LK2 V2 VDS1
7-9	Ghana/78	2	LK1 V1 VDS1
10-12	Nigeria/76	2	BK6 V3 VDS1
13-15	Ivory Coast/89	1	VDS1
16-18	Guinea-Bissau/91	1	V5 VDS1

^1^Cell types used were: CVS, CV1 expressing goat SLAM; VDS, Veros expressing dog SLAM; V, Vero; BK, primary bovine kidney; LK, primary lamb kidney.

### Animal studies

The animal study was carried out in the Pirbright institute high containment isolation units under the auspices of relevant UK legislation. The work was covered by a project licence issued by the UK Home Office and all staff carrying out procedures on animals had appropriate personal licences. The work was reviewed and approved by the Pirbright Institute Animal Welfare and Ethical Review Body. Outbred British male goats, 10-13 months in age, were infected with 10^5^ TCID_50_ of PPRV in a final volume of 1 mL, diluted in PBS where required (Kurdistan/2011, which only grew to a titre of 10^5^ TCID_50_/mL, was used undiluted). Three goats were inoculated with each PPRV isolate. The first two animals in each group were given the virus as a subcutaneous injection, while the third animal received the virus intranasally using a syringe-mounted Mucosal Atomiser Device (MAD 300, LMA, USA)). Blood samples were taken from a superficial vein into vacutainers prepared for serum or containing heparin or EDTA (Becton-Dickinson) or for subsequent RNA isolation (Tempus Blood RNA Tubes, Life Technologies). The rectal temperature of the animals was determined daily for 3 days prior to infection and for up to 21 days post infection (dpi). White cell counts were determined from anti-coagulant (EDTA)-treated blood.

### Other assays

Isolation of RNA from blood samples and real-time PCR assay of PPRV genome was carried out on anticoagulant (EDTA)-treated blood as previously described [16]. Isolation of RNA from blood collected in Tempus tubes and subsequent real-time PCR assay of goat cytokine and housekeeping gene mRNAs was carried out essentially as described [17], except that the primers and annealing temperatures used were those indicated in Table 2. The threshold cycle (Ct) for each assay for each animal was the mean of triplicates, and was normalised using the mean Ct for three housekeeping genes (glyceraldehyde phosphate dehydrogenase (GAPDH), succinate dehydrogenase A (SDHA) and glucose 6 phosphate dehydrogenase (G6PD)).

**Table 2:** PCR primer pairs and reaction conditions used in the work described in this paper

**Target**	**Primer sequences**	**Ta**^**1**^	**[Primer]**^**2**^	**Reference**
IL-1β	CCTTGGGTATCAGGGACAA	60	200 nM	This paper
	GGGTATGGCTTTCTTTAGG			
IL-4	ACCTGTTCTGTGAATGAAGCCAA	61	300 nM	This paper
	CTTCATAATAGTCTTTAGCCTTTC			
IL-6	TCCAGAACGAGTTTGAGG	60	400 nM	[18]
	CATCCGAATAGCTCTCAG			
IL-8	ATGAGTACAGAACTTCGA	52	300 nM	[18]
	TCATGGATCTTGCTTCTC			
IL-10	TGCTGTTGACCCAGTCTCTG	60	200 nM	[17]
	AGGGCAGAAAACGATGACAG			
IL-12A	GGGCATTGTCTGGCTTCTG	60	200 nM	This paper
	TTCTTCCAGGGAGGGTTTCT			
IL-12B	GCTGGGAGTACCCTGACACG	63	200 nM	[19]
	GTGACTTTGGCTGAGGTTTGGTC			
IL-18	ACTGTTCAGATAATGCACCCCAG	60	300 nM	[19]
	TTCTTACACTGCACAGAGATGGTTAC			
Interferon β	CCAGATGGTTCTCCTGCTGTGT	60	300 nM	[17]
	GACCAATACGGCATCTTCCTTC			
TNFα	GGAATACCTGGACTATGCTGA	60	300 nM	This paper
	CCTCACTTCCCTACATCCCT			
TGFβ	GGACATTAACGGGTTCAGTTC	57	200 nM	This paper
	GTCCAGGCTCCAGATGTAAG			
Interferon γ	CTCCGGCCTAACTCTCTCCT	60	300 nM	[17]
	AGGCCCACCCTTAGCTACAT			
GAPDH	GGTGATGCTGGTGCTGAGTA	60	300 nM	[18]
	TCATAAGTCCCTCCACGATG			
SDHA	ACCTGATGCTTTGTGCTCTGC	60	200 nM	[18]
	CCTGGACGGGCTTGGAGTAA			
G6PDH	CGAGGCTGTGTACACCAAGA	60	300 nM	[17]
	ATGTGGTGGAGCAGTGGAGT			

^1^Ta: annealing temperature.

^2^[Primer]: primer concentration.

For the determination of numbers of different blood cell types by flow cytometry, heparinised blood was stained with antibodies for surface markers CD4, CD8, CD14 and WC1. Live cells were stained using Live/dead Aqua (Life Technologies). Monoclonal antibody to CD4 was conjugated to allophycocyanin (APC) (clone 44.38, MCA2213A647 from Serotec) while anti-CD8 was conjugated to R-phycoerythrin (R-PE) (clone CC63, MCA837 from Serotec). Anti-CD14 (clone CCG33) and anti-WC1, a marker for γδ T cells (clone 197) were provided as hybridoma supernatant by the Microbiological Services division of the Pirbright Institute, and were directly conjugated to R-PE and APC respectively, using Zenon® kits (Life Technologies). Red blood cells were lysed using BD FACS Lysis solution (BD Biosciences). Labelled cells were fixed in 4% paraformaldehyde (PFA). Cells were analysed using the LSR Fortessa (BD Biosciences). The BD Biosciences DIVA software was used to acquire the data and FCS Express 3 (De Novo Software) or FlowJo (Tree Star Inc.) used for analysis. Changes in the composition of the lymphocytes were analysed for statistical significance using one-way ANOVA and Tukey’s correction for multiple comparisons (GraphPad Prism).

Statistical analysis of other data from the animal experiment was performed using a mixed model ANOVA as implemented in Minitab 16, with the virus used and the days post infection fixed factors and the animals in each group as random factors. The significance of any increase or decrease of transcription on day 2, 4 and 6, compared to the value at day zero, was determined using Dunnett’s correction for multiple comparisons.

## Results

We selected six isolates of PPRV that had either been stored lyophilised at the Institute soon after isolation or had been isolated from a recent outbreak. Each virus isolate was grown through one passage on Vero cells expressing the canine version of the generally conserved morbillivirus receptor, SLAM (CD150) [20]. Vero-dog-SLAM (VDS) cells [14] have proven to be a very good host for the growth of wild-type PPRV in our laboratory. The titre of each virus stock was determined on the same cell line. It should be noted that PPRV titres on VDS cells are at least 10 times higher than the titre of the same virus stock on unmodified Veros. Eighteen goats were infected in groups of three with one of the six isolates, listed in Table 1, and the animals monitored for up to 21 days, or until the pre-defined humane end point was reached. For the studies described here, each animal was compared to itself at day zero. One animal in each group was infected intranasally and the other two by the more conventional subcutaneous route. No difference was observed in the onset or severity of disease between the two routes of infection.

**Figure 1:**
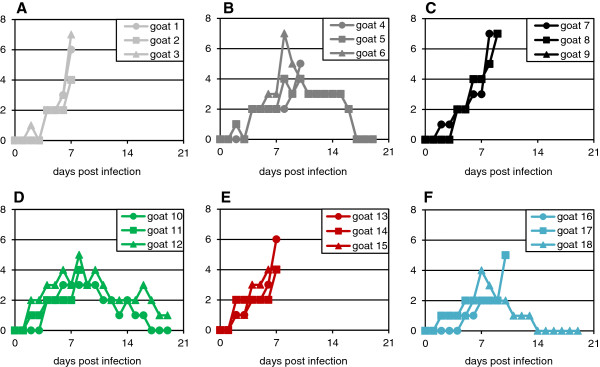
**Clinical scores of individual animals infected with different PPRV isolates.** Groups of three animals were inoculated as described in the text with 10^5^ TCID_50_ of **(A)** Kurdistan/2011, **(B)** Sudan/Sinnar/72, **(C)** Ghana/78, **(D)** Nigeria/76, **(E)** Ivory Coast/89, **(F)** Guinea-Bissau/91. The score for each animal was determined based on the observations recorded in Table 3.

**Figure 2:**
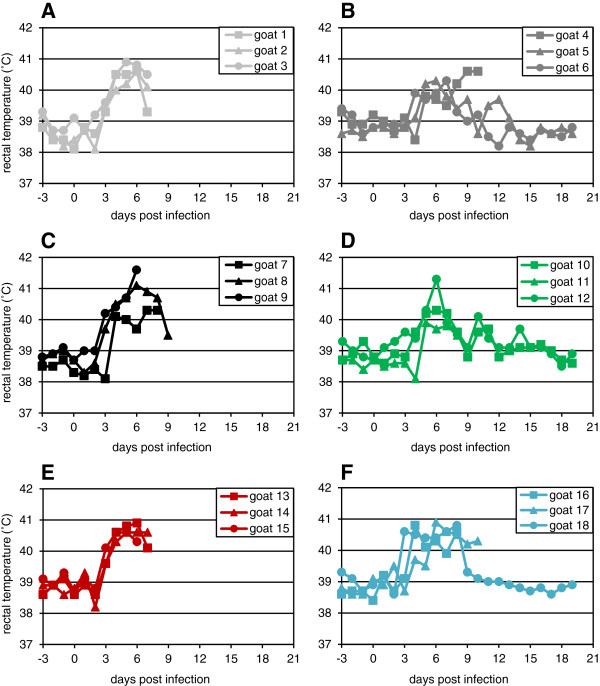
**Rectal temperature of individual animals infected with different PPRV isolates.** Groups of three animals were inoculated as described in the text with 10^5^ TCID_50_ of **(A)** Kurdistan/2011, **(B)** Sudan/Sinnar/72, **(C)** Ghana/78, **(D)** Nigeria/76, **(E)** Ivory Coast/89, **(F)** Guinea-Bissau/91. The temperature was determined daily, beginning 3 days before infection (to determine the baseline body temperature).

The infected goats in this study began to show clinical signs of disease, primarily congestion of the conjunctiva and/or nasal cavity, as early as 2 dpi (Figure 1); all the animals showed some ocular or nasal signs by 4 dpi (Table 3). Animals began to show nasal and/or ocular discharge at day 6. At 7 dpi, some animals began to reach the humane end point of the protocol licence, and had to be euthanised. Pyrexia was detectable in a few animals at 3 dpi, rising to a peak temperature in all animals at 5–6 dpi (Figure 2). Animals that survived past 9 dpi showed gradually decreasing pyrexia and other clinical signs.

**Table 3:** Days post infection when different clinical signs of disease were recorded on each goat

**Goat**	**Ocular**		**Nasal**		**Oral**	**Oral**	**Diarrhoea**	**Cough**	**Apathy**
	**Congestion**	**+discharge**	**Congestion**	**+discharge**	**Congestion**	**Lesions**			
**1**	4-6	7	4 5	6 7		7			6 7
**2**	4-7		4-7					7	6 7
**3**	2-6	7	2-5	6 7	7			7	6 7
**4**	4-10		4-10			10		6 8 9	
**5**	2-7 9 11-16	8 10	4-7 16	8-15				15	10-12
**6**	4-7 9-16	8	4 5 16	6-15	9	8	8 9	10	10
**7**	4-7	8	4-7	8			8	6-8	8
**8**	4-9		4 5	6-9	9		7 8	6 8 9	7
**9**	4-6		4 5	6			5 6		5 6
**10**	4-12		4 5 11-15	6-10				8 11 12	
**11**	2-8		4-7	8					5-8
**12**	2-17		2-7 11-19	8-10			8	4-8 10 11	
**13**	4-7		2-6	7			7	6 7	6 7
**14**	2-7		2-6	7				7	6 7
**15**	2-6		4 5	6	4-6				
**16**	5-8							7 8	6-8
**17**	2 5-10		5-9	10	10				10
**18**	6-10		6-13			7		8	

**Figure 3:**
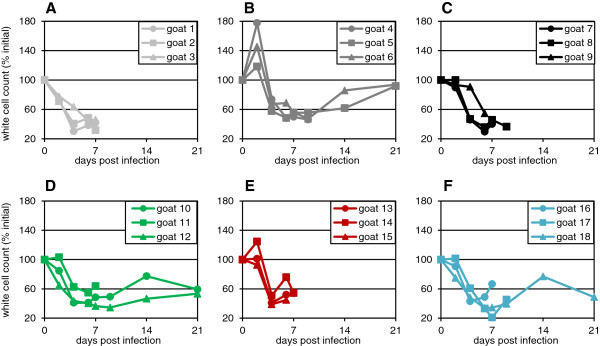
**White cell counts of individual animals infected with different PPRV isolates.** Groups of three animals were inoculated as described in the text with 10^5^ TCID_50_ of **(A)** Kurdistan/2011, **(B)** Sudan/Sinnar/72, **(C)** Ghana/78, **(D)** Nigeria/76, **(E)** Ivory Coast/89, **(F)** Guinea-Bissau/91. White cell counts in peripheral blood were determined on each day that blood was sampled and plotted as the percent of the initial (day 0) value to allow for variation between individual animals.

All the infected animals developed leukopenia to a greater or lesser degree, with up to 80% reduction in white cell counts in peripheral blood (Figure 3). Leukopenia was observed earlier than any other sign, some animals showing a clear decrease in white cell count at 2 dpi. The leukopenia persisted beyond the course of the study in animals that survived infection and which could therefore be monitored through to 21 dpi. This is in contrast to other clinical signs, which developed at the same time as or after the decrease in white cell count and which resolved more rapidly. There was a slight correlation between the degree of disease (clinical score) and the final loss of white cells, though it was not marked. Interestingly, all the animals infected with the Sudan/Sinnar/72 isolate showed an initial increase in white cell count at 2 dpi, but the white cell count then decreased to about 40% of the initial value with a similar time course to that seen in the other infected animals. It is not clear why this isolate induced such a marked effect on white cell count. However, it is possible that leucocytosis is also occurring in the other infected animals at this early stage of infection (e.g. a slight leucocytosis was seen in goat 14 infected with the otherwise pathogenic Ivory Coast/89 isolate), but that this has been hidden by the contrary effect (reduction in circulating white cells). The observations in the Sudan/Sinnar/72 group of animals are unlikely to be because this isolate had developed a high concentration of defective interfering (DI) particles in cell culture passage, as DIs would be likely to lead to a more rapid induction of type I interferon [21], which would result in a reduction, not an increase, of white cells [22-24]. It is also not a reaction to the culture medium containing the virus, as goats 1–3 received a much larger dose of medium (since this isolate grew to a lower titre than the other isolates) and these animals showed no such effect.

**Figure 4:**
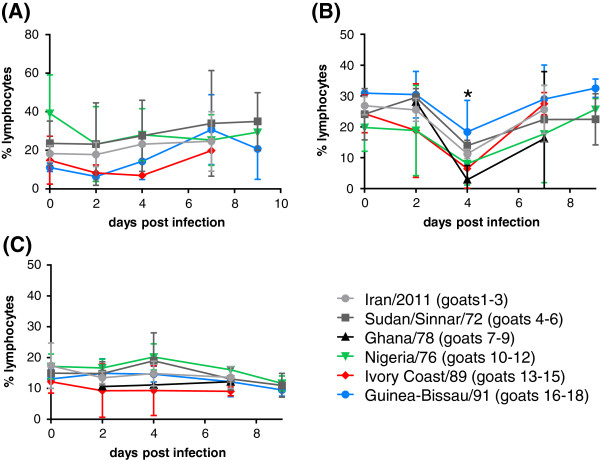
**Effects of PPRV infection on circulating immune cells.** Goats were infected with different PPRV isolates as described in the text. Whole blood was taken from infected animals at the indicated days post infection and was stained with monoclonal anti-CD4, anti-CD8 and anti-CD14 monoclonal antibodies. Results are expressed as the mean ± SD of the percentage of total lymphocytes that were **(A)** CD14^+^, **(B)** CD4^+^ or **(A)** CD8^+^ cells in peripheral blood. * = *p* < 0.01 (not different from day zero).

Examination by flow cytometry of the numbers of CD14^+^ monocytes and WC1^+^, CD4^+^ and CD8^+^ T cells in peripheral blood following infection showed only a transient reduction in the proportion of CD4^+^ cells at 4 dpi (Figure 4). No change was seen in the % of total lymphocytes that were CD8^+^ or CD14^+^ at any time point, whereas the % CD4^+^ decreased by about 60% at 4 dpi; this decrease was statistically significant (*p* < 0.01). The apparent recovery of the % of cells that were CD4^+^ cells at 7 dpi, in the absence of recovery of white cell count in general, suggests that there was an early loss of CD4^+^ cells followed by a subsequent loss of other cell types. This contrasts with a study of circulating lymphocytes in children infected with measles, where the CD4^+^/CD8^+^ ratio was still decreased in infected subjects at up to 1 month following infection [25]. No change was observed in this study in the percent of lymphocytes that were WC1^+^ (γ/δ T cells) between days 2 and 9 post infection, although data for this marker was not acquired on day 0 (not shown).

**Figure 5:**
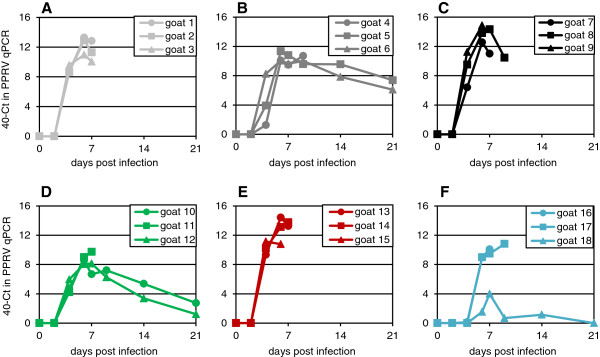
**PPRV-specific RNA detected in the blood of individual animals infected with different PPRV isolates.** Groups of three animals were inoculated as described in the text with 10^5^ TCID_50_ of **(A)** Kurdistan/2011, **(B)** Sudan/Sinnar/72, **(C)** Ghana/78, **(D)** Nigeria/76, **(E)** Ivory Coast/89, **(F)** Guinea-Bissau/91. Real-time PCR was carried on RNA extracted from whole blood as described in Methods. The real-time PCR values for each animal on each day are plotted as 40-(observed Ct).

The level of viraemia was estimated by real-time PCR for the PPRV genome. All the viruses, with the exception of the Guinea-Bissau/91 isolate, showed clear viraemia at 4 dpi, rising to a peak at 6–7 dpi (Figure 5). The viraemia appears to be prolonged, with virus still detectable in the blood up to 21 dpi, though again the Guinea-Bissau isolate appears to differ from the others tested, in that the one animal that survived (goat 18) showed only low levels of viral genome in the blood at any time point, and none at all at 21 dpi. Interestingly, this animal showed similar leukopenia, though a reduced clinical score profile and a delayed pyrexia, compared to other infected animals.

**Figure 6:**
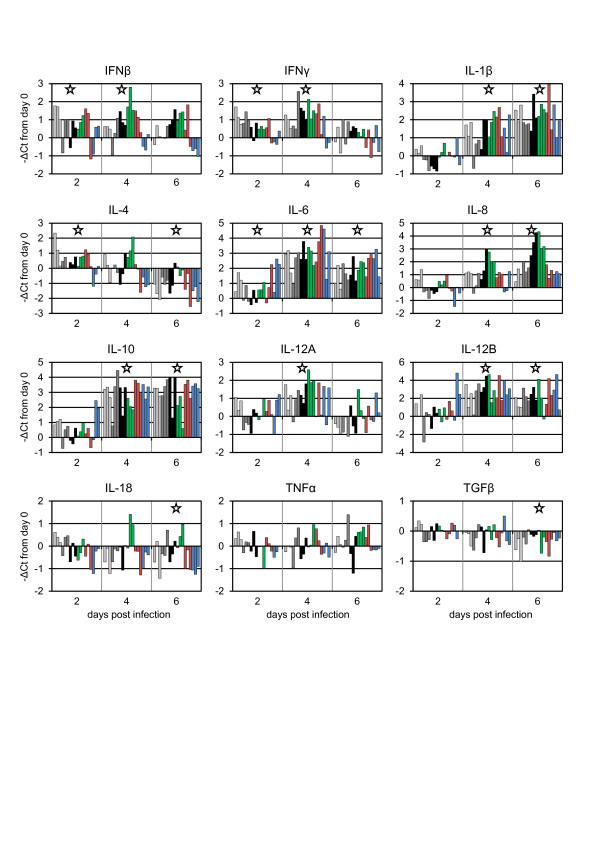
**Changes in cytokine mRNA transcription in goats infected with PPRV.** Animals were infected with different PPRV isolates as described in Figure 1. The changes in transcription of mRNA for various cytokines was determined for each experimental animal over the first six days of the study and are plotted as –ΔCt from day zero, so that an increase in mRNA appears as an increase in the value plotted. Stars indicate days on which the transcription of that cytokine was significantly different from day zero. Bars are colour-coded as in Figures 1–5 to indicate the different PPRV isolates used.

The effects of infection on the expression of various cytokines was assessed using real-time PCR assays targeted to caprine cytokine mRNA sequences (Table 2). Assays were carried out only for days 0–6 dpi when all animals were still alive and a complete data set was available. No significant difference was seen between virus isolates for any cytokine. No effect was seen on TNFα expression at any time point. There was a slight but significant decrease in transcription of IL-18 and TGFβ at 6 dpi (Figure 6). Transcription of IFNβ and IFNγ was raised at 2 dpi and 4 dpi, although transcription of both was falling again at 6 dpi, when clinical signs were still increasing in severity. Transcription of IL-4 was initially increased (at 2 dpi), but then fell to below normal levels by 6 dpi. This initial rise and then fall of transcription is similar to that previously reported for IL-4 in Indian hill goats infected with pathogenic PPRV [13]. IL-1β, IL-8 and IL-10 were increased at 4 dpi and 6 dpi, and IL-6 transcription was raised at all time points, although it appeared to be falling at 6 dpi, while transcription of IL-8 was still increasing at 6 dpi. Interestingly, IL-12A, the p37 subunit of IL-12, was clearly induced, as expected, at 4 dpi, but had fallen to near normal levels by 6 dpi. In contrast, the transcription of IL-12B (the p40 subunit of IL-12, which also functions as a component of IL-23) was still high at 6 dpi. IL-10 transcription was significantly increased by 4 dpi, which may explain the observed decrease in IL-12 expression, and hence also the consequent decrease in IFNγ expression [26].

## Discussion

Several recent papers have commented on a reputed difficulty in reproducing PPR disease in experimental animals (e.g. [10,27]), while other investigators have apparently found little difficulty in eliciting severe disease (e.g. [6,28,29]. One of the purposes of this study was to establish a clear model of PPRV disease in UK goats. Despite their different lineages and isolation date or route of infection, all the viruses used in this study induced similar clinical signs of disease, although there was enough variation in the degree of severity of the signs that some groups of animals had to be euthanised by 7 dpi, whereas in other groups one or two animals survived (Figure 1). Groups 1, 3 and 5 showed higher peak clinical scores (Figure 1) and higher peak viraemia (Figure 5) than groups 2, 4 and 6, and all the animals in these groups were euthanised by day 9. No difference was seen in the peak leukopenia (Figure 3) or pyrexia (Figure 2) between the two groups that were all euthanised by 9 dpi and those where some animals survived. The disease in the British goats appears therefore to be less variable than in West African dwarf goats [6], where disease varied from peracute (death in 5–6 days) to mild when animals were infected with a similar selection of isolates of different lineages. In that case, the authors speculated that severe disease may be linked to pre-existing inapparent infections such as heartwater disease [6], which would not be a problem with the animals used in this study.

A previous study [27] reported that, in Alpine goats, intranasal infection with a recent Moroccan isolate of PPRV was found to give more rapid onset and more severe disease than subcutaneous infection with the same isolate. In the current study, comparing across all PPRV isolates (12 animals infected subcutaneously and 6 animals infected intranasally), no statistically significant difference was seen in the clinical signs of disease induced by the two routes of infection. There are several possible reasons for the difference in the observations in the two studies. It is possible that differences in the method of intranasal delivery may affect exactly where in the nasopharyngeal tract the virus is absorbed and hence how rapidly the virus reaches the lymph tissue where it initially replicates [10]. It is also possible that there are differences in the rate of uptake of virus through the subcutaneous tissues of the Alpine and UK breeds of goats, or even differences in the preference of the Moroccan isolate for cells of the nasopharyngeal mucosa. It is clear from other previous studies that subcutaneous infection can lead to very rapid and severe PPR disease (e.g. [6]), and the observations in these different studies underline the necessity of comparing virus virulence between different host species, as well as comparison of different virus isolates in the same host species.

The early decrease of IL-12 expression may be related to the observed decrease in transcription of IFNγ at this time, since IL-12 is required for the production of IFNγ in NK cells [26,30,31] as well as the long-term maintenance of the T cell IFNγ response [32,33], while IL-12 synthesis is primed by IFNγ [34,35]. It may also be significant for the progression of disease that transcription of IL-12B was still high at 6 dpi, while transcription of the p37 subunit had fallen, suggesting that the infected animals may develop an excess of the p40 subunit, which would lead to formation of homodimers of this protein. Such homodimers have been shown to act as antagonists of IL-12 (reviewed in [36]), and this may have an additional modulating action on the immediate immune response to infection. It should be noted that studies on the related morbillivirus measles virus have shown that IL-12 production is inhibited during and after infection with this virus [37-39]. Inhibition of IL-12 induction has been proposed as being responsible for the immunosuppression seen in all morbillivirus infections, although this inhibition appears to be only one element in a multifactorial effect [40].

The relatively early increase in IL-10 transcription may also be playing a role in PPR pathogenesis. The role of IL-10 in disease differs for different viruses. IL-10 production is observed only at later stages in mice infected with respiratory syncytial virus (RSV), and its expression is associated with viral clearance, while the absence of IL-10 leads to more severe disease [41]. In contrast, acute infection of mice with influenza A virus is enhanced by IL-10, and clearance enhanced by its absence [42]; similarly, IL-10 is important for the pathogenesis of West Nile virus [43], and is associated with pathogenic disease caused by Dengue virus [44]. Mistimed IL-10 responses can inhibit the pro-inflammatory response, preventing the natural defence systems from clearing infecting pathogens (reviewed in [45]); increased levels of IL-10 may therefore contribute to the general immune suppression seen in PPRV infection. Strong induction of IL-10 by 4 dpi was also seen in sheep infected with pathogenic, but not apathogenic, Nairobi sheep disease virus [17]. It is not possible to say at this stage whether the observed induction of IL-10 is a cause or consequence of the pathogenic effect of virus infection in these animals. In the study reported here, we did notice that the isolate of PPRV that caused the least disease in these studies (Guinea-Bissau/91) showed as strong an induction of IL-10 as the other isolates, which would argue against disease in PPRV infection being specifically associated with IL-10 expression, but rather that this is part of the normal defensive response. In fact, none of the cytokines whose expression in blood cells was measured showed any distinction between the more pathogenic viruses (groups 1, 3 and 5) and the less pathogenic (groups 2, 4 and 6), suggesting that all the virus isolates are causing essentially the same disease, with the difference being one of degree rather than qualitative. It will be very interesting to compare these observations with similar studies of PPRV infection in animals such as cattle which normally do not show disease when exposed to this virus.

No significant difference was seen in clinical signs, viraemia or cytokine induction between individual lineages of PPRV. Where two viruses from the same lineage were used (group 3 vs 4 and group 5 vs 6), in each case one isolate was of the more pathogenic group and one from the less pathogenic. It seems likely that individual isolates from any lineage may be more or less pathogenic. The clearest difference between the isolates that showed the most pathogenicity in this study and those that elicited a milder disease was the number of prior passages in cell culture, with the less pathogenic isolates having at least 5 passages in non-SLAM bearing cells. This suggests that it would be advisable for comparisons of the virulence of different PPRV strains to take into account even small differences in the passage history of the isolate.

## Competing interests

The authors declare that they have no competing interests.

## Authors’ contributions

JB prepared the viruses and processed samples from the animal study. JB and ABT carried out the real-time PCR studies of the goat cytokines. RH carried out the FACS analysis. LF carried out the real-time PCR of the viral RNA. GT and MDB conceived, designed and directed the study. MDB carried out animal work and prepared the manuscript. All authors read and approved the manuscript.

## References

[B1] Diallo A Control of peste des petits ruminants and poverty alleviation? J Vet Med B Infect Dis Vet Public Health 2006 53 Suppl 1 11 1317123358

[B2] Banyard AC Parida S Batten C Oura C Kwiatek O Libeau G Global distribution of peste des petits ruminants virus and prospects for improved diagnosis and control J Gen Virol 2010 91 2885 2897 10.1099/vir.0.025841-0 20844089

[B3] Baron MD Parida S Oura CA Peste des petits ruminants: a suitable candidate for eradication? Vet Rec 2011 169 16 21 10.1136/vr.d3947 21724765

[B4] Albina E Kwiatek O Minet C Lancelot R Servan de Almeida R Libeau G Peste des Petits Ruminants, the next eradicated animal disease? Vet Microbiol 2013 165 38 44 10.1016/j.vetmic.2012.12.013 23313537

[B5] Wohlsein P Saliki J Barrett T, Pastoret P-P, Taylor WP Rinderpest and peste des petits ruminants - the diseases: clinical signs and pathology Rinderpest and Peste des Petits Ruminants 2006 London, Burlington, San Diego: Academic 68 85 [Pastoret P-P (Series Editor): *Biology of Animal Infections*]

[B6] Couacy-Hymann E Bodjo C Danho T Libeau G Diallo A Evaluation of the virulence of some strains of peste-des-petits-ruminants virus (PPRV) in experimentally infected West African dwarf goats Vet J 2007 173 178 183 10.1016/j.tvjl.2005.08.020 16310383

[B7] Lefèvre PC Diallo A Peste des petits ruminants Rev Sci Tech 1990 9 935 981 2132714 10.20506/rst.9.4.532

[B8] Singh RP Saravanan P Sreenivasa BP Singh RK Bandyopadhyay SK **Prevalence and distribution of peste des petits ruminants virus infection in small ruminants in India** Rev Sci Tech 2004 23 807 819 15861876 10.20506/rst.23.3.1522

[B9] Delil F Asfaw Y Gebreegziabher B Prevalence of antibodies to peste des petits ruminants virus before and during outbreaks of the disease in Awash Fentale district, Afar, Ethiopia Trop Anim Health Prod 2012 44 1329 1330 10.1007/s11250-012-0110-8 22359089

[B10] Pope RA Parida S Bailey D Brownlie J Barrett T Banyard AC Early events following experimental infection with peste-des-petits ruminants virus suggest immune cell targeting PLoS One 2013 8 e55830 10.1371/journal.pone.0055830 23418464 PMC3572172

[B11] Lemon K de Vries RD Mesman AW McQuaid S van Amerongen G Yuksel S Ludlow M Rennick LJ Kuiken T Rima BK Geijtenbeek TB Osterhaus AD Duprex WP de Swart RL Early target cells of measles virus after aerosol infection of non-human primates PLoS Pathog 2011 7 e1001263 10.1371/journal.ppat.1001263 21304593 PMC3029373

[B12] de Vries RD Lemon K Ludlow M McQuaid S Yuksel S van Amerongen G Rennick LJ Rima BK Osterhaus AD de Swart RL Duprex WP In vivo tropism of attenuated and pathogenic measles virus expressing green fluorescent protein in macaques J Virol 2010 84 4714 4724 10.1128/JVI.02633-09 20181691 PMC2863733

[B13] Patel A Rajak KK Balamurugan V Sen A Sudhakar SB Bhanuprakash V Singh RK Pandey AB Cytokines expression profile and kinetics of Peste des petits ruminants virus antigen and antibody in infected and vaccinated goats Virol Sin 2012 27 265 271 10.1007/s12250-012-3240-2 22899436 PMC8218146

[B14] Seki F Ono N Yamaguchi R Yanagi Y Efficient isolation of wild strains of canine distemper virus in Vero cells expressing canine SLAM (CD150) and their adaptability to marmoset B95a cells J Virol 2003 77 9943 9950 10.1128/JVI.77.18.9943-9950.2003 12941904 PMC224612

[B15] Vilcek S Herring AJ Herring JA Nettleton PF Lowings JP Paton DJ Pestiviruses isolated from pigs, cattle and sheep can be allocated into at least three genogroups using polymerase chain reaction and restriction endonuclease analysis Arch Virol 1994 136 309 323 10.1007/BF01321060 8031236

[B16] Batten CA Banyard AC King DP Henstock MR Edwards L Sanders A Buczkowski H Oura CC Barrett T A real time RT-PCR assay for the specific detection of Peste des petits ruminants virus J Virol Methods 2011 171 401 404 10.1016/j.jviromet.2010.11.022 21126540

[B17] Bin Tarif A Lasecka L Holzer B Baron MD Ganjam virus/Nairobi sheep disease virus induces a pro-inflammatory response in infected sheep Vet Res 2012 43 71 10.1186/1297-9716-43-71 23083136 PMC3507801

[B18] Smeed JA Watkins CA Rhind SM Hopkins J Differential cytokine gene expression profiles in the three pathological forms of sheep paratuberculosis BMC Vet Res 2007 3 18 10.1186/1746-6148-3-18 17697353 PMC1994670

[B19] Li H Cunha CW Davies CJ Gailbreath KL Knowles DP Oaks JL Taus NS Ovine herpesvirus 2 replicates initially in the lung of experimentally infected sheep J Gen Virol 2008 89 1699 1708 10.1099/vir.0.2008/000554-0 18559941

[B20] Tatsuo H Ono N Tanaka K Yanagi Y SLAM (CDw150) is a cellular receptor for measles virus Nature 2000 406 893 897 10.1038/35022579 10972291

[B21] Strahle L Garcin D Kolakofsky D Sendai virus defective-interfering genomes and the activation of interferon-beta Virology 2006 351 101 111 10.1016/j.virol.2006.03.022 16631220

[B22] Degre M Influence of exogenous interferon on the peripheral white blood cell count in mice Int J Cancer 1974 14 699 703 10.1002/ijc.2910140602 4463167

[B23] Hawkins MJ Krown SE Borden EC Krim M Real FX Edwards BS Anderson SA Cunningham-Rundles S Oettgen HF American cancer society Phase I trial of naturally produced beta-interferon Cancer Res 1984 44 5934 5938 6498851

[B24] Schattner A Meshorer A Wallach D Involvement of interferon in virus-induced lymphopenia Cell Immunol 1983 79 11 25 10.1016/0008-8749(83)90046-1 6861209

[B25] Ryon JJ Moss WJ Monze M Griffin DE Functional and phenotypic changes in circulating lymphocytes from hospitalized zambian children with measles Clin Diagn Lab Immunol 2002 9 994 1003 12204949 10.1128/CDLI.9.5.994-1003.2002 PMC120077

[B26] D’Andrea A Aste-Amezaga M Valiante NM Ma X Kubin M Trinchieri G Interleukin 10 (IL-10) inhibits human lymphocyte interferon gamma-production by suppressing natural killer cell stimulatory factor/IL-12 synthesis in accessory cells J Exp Med 1993 178 1041 1048 10.1084/jem.178.3.1041 8102388 PMC2191152

[B27] El Harrak M Touil N Loutfi C Hammouchi M Parida S Sebbar G Chaffai N Harif B Messoudi N Batten C Oura CA A reliable and reproducible experimental challenge model for peste des petits ruminants virus J Clin Microbiol 2012 50 3738 3740 10.1128/JCM.01785-12 22915602 PMC3486268

[B28] Kumar P Tripathi BN Sharma AK Kumar R Sreenivasa BP Singh RP Dhar P Bandyopadhyay SK Pathological and immunohistochemical study of experimental peste des petits ruminants virus infection in goats J Vet Med B Infect Dis Vet Public Health 2004 51 153 159 10.1111/j.1439-0450.2004.00747.x 15228548

[B29] Couacy-Hymann E Bodjo SC Danho T Koffi MY Libeau G Diallo A Early detection of viral excretion from experimentally infected goats with peste-des-petits ruminants virus Prev Vet Med 2007 78 85 88 10.1016/j.prevetmed.2006.09.003 17064800

[B30] Orange JS Biron CA An absolute and restricted requirement for IL-12 in natural killer cell IFN-gamma production and antiviral defense. Studies of natural killer and T cell responses in contrasting viral infections J Immunol 1996 156 1138 1142 8557990

[B31] Gazzinelli RT Hieny S Wynn TA Wolf S Sher A Interleukin 12 is required for the T-lymphocyte-independent induction of interferon gamma by an intracellular parasite and induces resistance in T-cell-deficient hosts Proc Natl Acad Sci U S A 1993 90 6115 6119 10.1073/pnas.90.13.6115 8100999 PMC46878

[B32] John B Rajagopal D Pashine A Rath S George A Bal V Role of IL-12-independent and IL-12-dependent pathways in regulating generation of the IFN-gamma component of T cell responses to Salmonella typhimurium J Immunol 2002 169 2545 2552 12193724 10.4049/jimmunol.169.5.2545

[B33] Yap G Pesin M Sher A Cutting edge: IL-12 is required for the maintenance of IFN-gamma production in T cells mediating chronic resistance to the intracellular pathogen, Toxoplasma gondii J Immunol 2000 165 628 631 10878333 10.4049/jimmunol.165.2.628

[B34] Fantuzzi L Gessani S Borghi P Varano B Conti L Puddu P Belardelli F Induction of interleukin-12 (IL-12) by recombinant glycoprotein gp120 of human immunodeficiency virus type 1 in human monocytes/macrophages: requirement of gamma interferon for IL-12 secretion J Virol 1996 70 4121 4124 8648753 10.1128/jvi.70.6.4121-4124.1996 PMC190299

[B35] Ma X Chow JM Gri G Carra G Gerosa F Wolf SF Dzialo R Trinchieri G The interleukin 12 p40 gene promoter is primed by interferon gamma in monocytic cells J Exp Med 1996 183 147 157 10.1084/jem.183.1.147 8551218 PMC2192398

[B36] Holscher C The power of combinatorial immunology: IL-12 and IL-12-related dimeric cytokines in infectious diseases Med Microbiol Immunol 2004 193 1 17 10.1007/s00430-003-0186-x 12836019

[B37] Karp CL Wysocka M Wahl LM Ahearn JM Cuomo PJ Sherry B Trinchieri G Griffin DE Mechanism of suppression of cell-mediated immunity by measles virus Science 1996 273 228 231 10.1126/science.273.5272.228 8662504

[B38] Atabani SF Byrnes AA Jaye A Kidd IM Magnusen AF Whittle H Karp CL Natural measles causes prolonged suppression of interleukin-12 production J Infect Dis 2001 184 1 9 10.1086/321009 11398102

[B39] Polack FP Hoffman SJ Moss WJ Griffin DE Altered synthesis of interleukin-12 and type 1 and type 2 cytokinesin rhesus macaques during measles and atypical measles J Infect Dis 2002 185 13 19 10.1086/338009 11756976

[B40] Hoffman SJ Polack FP Hauer DA Singh M Billeter MA Adams RJ Griffin DE Vaccination of rhesus macaques with a recombinant measles virus expressing interleukin-12 alters humoral and cellular immune responses J Infect Dis 2003 188 1553 1561 10.1086/379250 14624382

[B41] Loebbermann J Schnoeller C Thornton H Durant L Sweeney NP Schuijs M O’Garra A Johansson C Openshaw PJ IL-10 regulates viral lung immunopathology during acute respiratory syncytial virus infection in mice PLoS One 2012 7 e32371 10.1371/journal.pone.0032371 22393401 PMC3290561

[B42] Sun K Torres L Metzger DW A detrimental effect of interleukin-10 on protective pulmonary humoral immunity during primary influenza A virus infection J Virol 2010 84 5007 5014 10.1128/JVI.02408-09 20200252 PMC2863832

[B43] Bai F Town T Qian F Wang P Kamanaka M Connolly TM Gate D Montgomery RR Flavell RA Fikrig E IL-10 signaling blockade controls murine West Nile virus infection PLoS Pathog 2009 5 e1000610 10.1371/journal.ppat.1000610 19816558 PMC2749443

[B44] Malavige GN Huang LC Salimi M Gomes L Jayaratne SD Ogg GS Cellular and cytokine correlates of severe dengue infection PLoS One 2012 7 e50387 10.1371/journal.pone.0050387 23209731 PMC3510251

[B45] Couper KN Blount DG Riley EM IL-10: the master regulator of immunity to infection J Immunol 2008 180 5771 5777 18424693 10.4049/jimmunol.180.9.5771

